# Integrated analysis of epigenomic and genomic changes by DNA methylation dependent mechanisms provides potential novel biomarkers for prostate cancer

**DOI:** 10.18632/oncotarget.2313

**Published:** 2014-08-06

**Authors:** Nicole M. A. White-Al Habeeb, Linh T. Ho, Ekaterina Olkhov-Mitsel, Ken Kron, Vaijayanti Pethe, Melanie Lehman, Lidija Jovanovic, Neil Fleshner, Theodorus van der Kwast, Colleen C. Nelson, Bharati Bapat

**Affiliations:** ^1^ Lunenfeld-Tanenbaum Research Institute, Mount Sinai Hospital; ^2^ Department of Laboratory Medicine and Pathobiology, University of Toronto; ^3^ Ontario Cancer Institute, Princess Margaret Cancer Center, University Health Network, Toronto, Canada; ^4^ Australian Prostate Cancer Research Center, Queensland University of Technology, Brisbane, Australia; ^5^ Department of Pathology, University Health Network, Toronto, Canada

**Keywords:** Prostate cancer, tumor markers, biomarkers, epigenetics, DNA methylation, expression array, DAC treatment, demethylating agent treatment

## Abstract

Epigenetic silencing mediated by CpG methylation is a common feature of many cancers. Characterizing aberrant DNA methylation changes associated with tumor progression may identify potential prognostic markers for prostate cancer (PCa). We treated two PCa cell lines, 22Rv1 and DU-145 with the demethylating agent 5-Aza 2’–deoxycitidine (DAC) and global methylation status was analyzed by performing methylation-sensitive restriction enzyme based differential methylation hybridization strategy followed by genome-wide CpG methylation array profiling. In addition, we examined gene expression changes using a custom microarray. Gene Set Enrichment Analysis (GSEA) identified the most significantly dysregulated pathways. In addition, we assessed methylation status of candidate genes that showed reduced CpG methylation and increased gene expression after DAC treatment, in Gleason score (GS) 8 vs. GS6 patients using three independent cohorts of patients; the publically available The Cancer Genome Atlas (TCGA) dataset, and two separate patient cohorts. Our analysis, by integrating methylation and gene expression in PCa cell lines, combined with patient tumor data, identified novel potential biomarkers for PCa patients. These markers may help elucidate the pathogenesis of PCa and represent potential prognostic markers for PCa patients.

## INTRODUCTION

Prostate cancer (PCa) is the most common urological malignancy in western countries. The American Cancer Society estimates that 233,000 American men will be diagnosed with PCa in 2014 [1]. In recent years, prostate specific antigen (PSA) testing has led to a sharp increase in PCa incidence [2], yet serum PSA does not accurately measure the threat that PCa poses to the patient's life [3]. It is estimated that approximately 15-30% of patients who undergo radical prostatectomy have been over-treated as the tumors would likely not affect their life span or quality of life [4], thus suffering the harsh side effects of this treatment without any clinical benefit. Tumors with the same histopathologic grade are biologically diverse, as some may remain indolent while others behave aggressively, leading to local recurrence and metastatic disease. Based on the vast tumor heterogeneity in PCa, more accurate biomarkers are needed to help accurately distinguish between indolent and aggressive PCas. A better understanding of the molecular mechanisms of PCa progression would help address the unmet clinical need for the identification of biomarkers that can accurately distinguish between indolent and aggressive tumors and would also help form the basis for the development of novel therapeutic targets.

Epigenetic changes, including DNA methylation, have been shown to play a role in prostate carcinogenesis by regulating gene expression. DNA methylation occurs when a methyl group is added to a cytosine base that precedes a guanine (CpG). This epigenetic change has been shown to play a role in promoting chromosomal stability and regulating gene expression [5, 6]. Aberrant DNA methylation patterns are the most widely studied epigenetic mechanism and have been shown to be valuable diagnostic [7-9], prognostic [10, 11], and predictive [12, 13] biomarkers for PCa and other cancers [14, 15]. We, and others have previously identified *HOXD3*, *TGFβ2*, *APC*, and *TBX15* as markers of PCa progression [16-20].

The integrated analyses, described in this manuscript, provide a comprehensive approach to identify potential novel candidate genes and signaling pathways that are regulated by epigenetic mechanisms and contribute to PCa progression. We compared genome wide methylation levels before and after treatment with the demethylating agent 5-Aza 2’ –deoxycytidine (DAC) to identify regions of CpG methylation in the PCa cell lines 22Rv1 and DU-145. We identified genes regulated by methylation mechanisms by determining differential gene expression post-DAC treatment in the same cells. Gene set enrichment analysis (GSEA) identified biological pathways that are over represented in these gene sets, including DNA replication and activation of ataxia telangiectasia and Rad3 related (ATR) in response to stress. Furthermore, we determined the prognostic significance of these markers by examining methylation levels in three independent cohorts of patients; two independent PCa patient cohorts with Gleason score (GS) 8 vs. GS6 tumors recruited at the University Health Network (UHN) and a third publically available dataset of PCa patients from The Cancer Genome Atlas (TCGA) database. These analyses identified novel potential methylation biomarkers associated with PCa progression.

## RESULTS

### Identification of methylated CpG probes in prostate cancer cell lines

**Figure 1:**
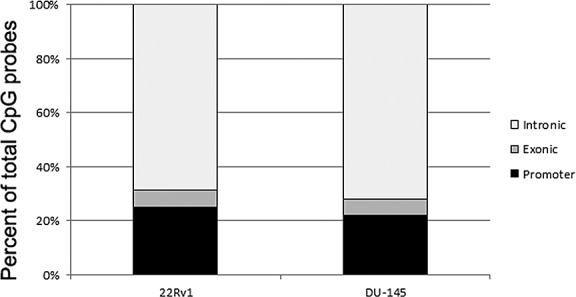
Bar graph showing the distribution of the location of CpG probes that were identified in the hypomethylated DNA fraction after treatment with 5-Aza 2’–deoxycitidine (DAC) In the 22Rv1 cells, we identified 11,212 probes with reduced methylation, representing 4922 genes (p<0.05) post-DAC treatment. In the DU-145 cells, we identified 32,511 CpG probes (representing 8008 genes, p<0.05) that showed reduced methylation after treatment. Interestingly, both cell lines showed similar distribution patterns of CpG locations. In the 22Rv1 cells, 25% CpG probes were found in promoter regions while 69% probes were located intragenically and 6% were located in intergenic regions. In the DU-145 cells, 22% CpG probes were found in promoter regions while 72% probes were located intragenically and 6% were located in intergenic regions.

We treated two cell lines with the demethylating agent 5-Aza 2’–deoxycytidine (DAC). 22Rv1 is derived from a PCa xenograft and is AR positive; while DU-145 is derived from PCa brain metastases and is AR negative. Global methylation status was analyzed by performing methylation-sensitive restriction enzyme based differential methylation hybridization strategy followed by genome-wide CpG methylation profiling. The Agilent Human CpG Island Microarray assessed genome-wide, 27,800 CpG islands with 237,220 probes in or within 95bp of CpG islands. The hypomethylated genomic DNA (gDNA) fractions were isolated and we compared CpG methylation status before and after treatment with DAC. In the 22Rv1 cells, we identified 11,212 CpG probes (representing 4922 genes; p<0.05) with reduced methylation post-DAC treatment, suggesting these probes were methylated in the untreated cells. We observed 25% identified CpG probes were found in promoter regions while 69% probes were intronic and 6% were located in intergenic regions (Figure 1). A representative list of 25 such methylated CpGs in 22Rv1 cells is shown in Table 2. The most significant hypomethylated region identified after DAC treatment was an intragenic region of tumor protein D52 (TPD52; p<0.001).

**Table 1:** Primer and probes sequences for MethyLight analysis

Gene	Forward Primer	Reverse Primer	Probe
*ACTA1*	5′-GGATTTTTTA GTGGGGTTTCGCG-3′	5′-CCAAAAACC TAAAAACATCTCC TACCG-3′	5′-AGGTCGAGAAGAGAATTTT TGGTCGTCGTTTTGGTAG-3′
*B4GALNT1*	5′-GTTTTGTAGGG GTGAAGCG -3′	5′-AATTACCTCCA AACGAACCTAA -3′	5′-AGGTATCGGAGCGTAGAT TTTGATTTTTTCGGGT-3′
*ALU-C4*	5′-GGTTAGGTATAGT GGTTTATATTTGTAATTT TAGTA-3′	5′-ATTAACTAAAC TAATCTTAAACTCCTA ACCTCA-3′	5′-CCTACCTTAACCTCCC-3′

**Table 2:** A representative list of CpG sites that showed significant increased hypomethylation in 22Rv1 cells after treatment with 5-Aza 2′ –deoxycitidine (DAC)

Gene Symbol	Gene Name	CpG Location	p value
*TPD52*	tumor protein D52	Intragenic	3.88E-07
*CDR2L*	cerebellar degeneration-related protein 2-like	Intragenic	1.50E-03
*GPER1*	G Protein-Coupled Estrogen Receptor 1	Promoter	2.00E-03
*CLK2*	CDC-like kinase 2	Intragenic	3.40E-03
*ADAM8*	ADAM metallopeptidase domain 8	Promoter	6.90E-03
*SSTR1*	somatostatin receptor 1	Intragenic	9.50E-03
*OSR1*	odd-skipped related 1	Intragenic	1.05E-02
*BANK1*	B-cell scaffold protein with ankyrin repeats 1	Promoter	1.08E-02
*ACTA1*	actin, Alpha 1, Skeletal Muscle	Intragenic	1.18E-02
*INPP5A*	inositol polyphosphate-5-phosphatase, 40kDa	Intragenic	1.78E-02
*CYP26A1*	cytochrome P450, family 26, subfamily A, polypeptide 1	Promoter	2.08E-02
*ADRA1A*	adrenoceptor alpha 1A	Promoter	2.26E-02
*TFDP1*	transcription factor Dp-1	Intragenic	2.28E-02
*RAB11B*	RAB11B, member RAS oncogene family 1	Intragenic	2.36E-02
*OBSCN*	obscurin, cytoskeletal calmodulin and titin-interacting RhoGEF	Intragenic	2.95E-02
*NCOR2*	nuclear receptor corepressor 2	Intragenic	2.99E-02
*TLX1*	T-cell leukemia homeobox 1	Intragenic	3.10E-02
*SCAND1*	SCAN domain containing 1	Promoter	3.38E-02
*KCND2*	potassium voltage-gated channel, Shal-related subfamily,member 2	Intragenic	3.64E-02
*DHRS12*	dehydrogenase/reductase (SDR family) member 12	Promoter	4.19E-02
*ONECUT2*	one cut homeobox 2	Intragenic	4.43E-02
*B4GALNT1*	beta-1,4-N-Acetyl-Galactosaminyl Transferase 1	Intragenic	4.47E-02
*CD248*	CD248 molecule, endosialin	Intergenic	4.48E-02
*HSF4*	heat shock transcription factor 4	Promoter	4.55E-02
*IRX3*	iroquois homeobox 3	Promoter	4.61E-02

We performed a parallel analysis with DU-145 cells and found 32,511 CpG probes (8008 genes; p<0.05) showed reduced methylation after treatment with DAC. 22% identified CpG probes were found in promoter regions while 72% probes were located intragenically and 6% were in intergenic regions (Figure 1). This pattern of differentially methylated CpG distribution throughout the genome is similar to the pattern observed in the 22Rv1 cells. A representative list of CpG probes identified with reduced methylation after DAC treatment in DU-145 cells is shown in Table 3. The top significant hypomethylated region identified after treatment was located in an intragenic region of the contactin associated protein-like 5 (CNTNAP5) gene. Other genes identified include glutathione S-transferase pi 1 (*GSTP1*), whose methylation in PCa is well documented in the literature, and homeobox D3 (*HOXD3*), T-box 15 (*TBX15*), and cytochrome P450, family 26, subfamily A, polypeptide 1 (*CYP26A1*), which we have previously shown to be methylated in PCa [21].

**Table 3:** A representative list of CpG sites that showed significant increased hypomethylation in DU-145 cells after treatment with 5-Aza 2′ –deoxycitidine (DAC)

Gene Symbol	Gene Name	CpG Location	p value
*CNTNAP5*	contactin associated protein-like 5	Intragenic	4.74E-06
*KIAA0415*	adaptor-related protein complex 5, zeta 1 subunit	Intragenic	1.00E-04
*SOGA2*	SOGA Family Member 2	Intragenic	7.00E-04
*ZNF418*	zinc finger protein 418	Intragenic	1.70E-03
*TBX15*	T-box 15	Intragenic	1.80E-03
*B4GALNT1*	Beta-1,4-N-Acetyl-Galactosaminyl Transferase 1	Intragenic	1.90E-03
*CENPM*	centromere protein M	Promoter	3.30E-03
*FIGN*	fidgetin	Promoter	3.30E-03
*ARRDC2*	arrestin domain containing 2	Promoter	3.30E-03
*OBSCN*	obscurin, cytoskeletal calmodulin and titin-interacting RhoGEF	Intragenic	3.50E-03
*HOXD3*	homeobox D3	Intragenic	3.70E-03
*MMP17*	matrix metallopeptidase 17 (membrane-inserted)	Intragenic	5.30E-03
*CRB2*	crumbs homolog 2 (Drosophila)	Intragenic	5.90E-03
*MARK3*	MAP/microtubule affinity-regulating kinase 3	Intragenic	1.71E-02
*CYP26A1*	cytochrome P450, family 26, subfamily A, polypeptide 1	Intragenic	2.23E-02
*GSTP1*	glutathione S-transferase pi 1	Intragenic	2.66E-02
*PDZD4*	PDZ domain containing 4	Intragenic	3.12E-02
*KLK12*	kallikrein-related peptidase 12	Intragenic	3.19E-02
*IRX2*	iroquois homeobox 2	Intragenic	3.31E-02
*ADCYAP1*	adenylate cyclase activating polypeptide 1 (pituitary)	Intragenic	3.37E-02
*MEX3B*	mex-3 RNA binding family member B	Intragenic	3.48E-02
*GPR135*	G protein-coupled receptor 135	Intragenic	3.49E-02
*COL9A3*	collagen, type IX, alpha 3	Intragenic	3.99E-02
*MAST1*	microtubule associated serine/threonine kinase 1	Intragenic	4.57E-02
*ACTA1*	actin, Alpha 1, Skeletal Muscle	Promoter	4.88E-02

In addition, we examined the common CpG regions that showed decreased methylation after DAC treatment in both the 22Rv1 and DU-145 cell lines. These genes may represent common pathways in PCa that are regulated by methylation mechanisms. We found there were 7406 CpG probes, representing 3380 unique genes that showed decreased methylation in both 22Rv1 and DU-145 cell lines after DAC treatment. Pathway analyses showed that these genes belong to pathways involved in the regulation of DNA methylation and histone modifications as well as the regulation of cell-cell adhesion and epithelial to mesenchymal transition, among others, which are important for tumorigenesis.

### Differential mRNA expression after DAC treatment

**Figure 2:**
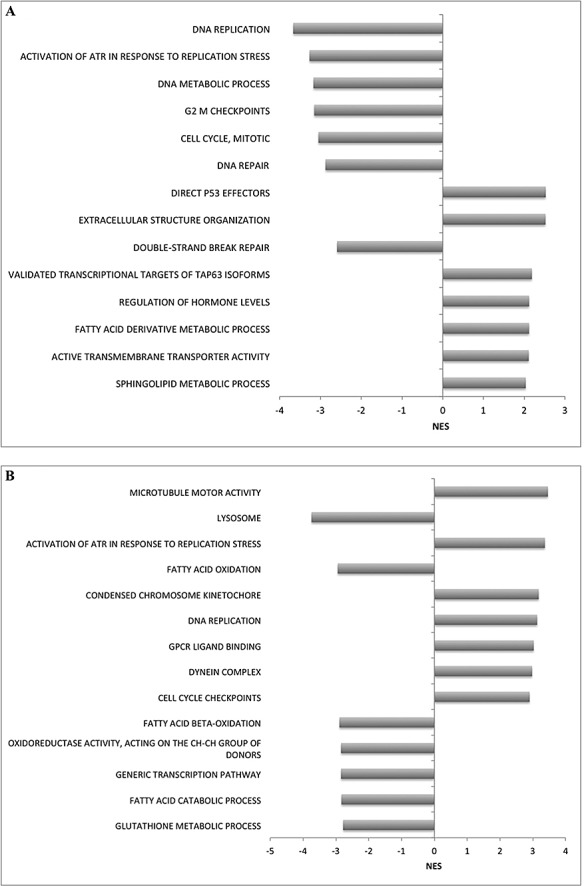
Enriched gene sets identified in (A) 22Rv1 and (B) DU-145 cells for upregulated genes following DAC treatment (positive normalized enrichment score (NES)) and downregulated genes post-DAC treatment (negative NES) Gene sets were found to be over-represented using GSEA software, with FDR (q-val) <0.05.

In order to investigate the contribution of DNA methylation to the regulation of gene expression in PCa, we treated cells with DAC and performed mRNA expression profiling. Gene expression was compared before and after treatment and was assayed using a custom microarray (GEO platform accession GPL16604), enriched for PCa metastasis-associated genes. We found 6598 probes (representing 2581 genes; FDR p<0.05) showed increased expression in 22Rv1 cells treated with DAC when compared to the untreated cells, suggesting expression of these genes may be, in part, regulated by methylation mechanisms. We also found there were 4604 probes (2020 genes; FDR p<0.05) that showed decreased expression in the treated cells. This may be due to either off target or indirect effects (e.g. demethylating a repressive factor, thereby increasing its expression and the repression of downstream targets). We performed GSEA pathway enrichment analysis to identify significant biological processes over represented in the 22Rv1 cells post-DAC treatment. We found that genes differentially regulated post-DAC treatment were most significantly enriched for DNA replication, activation of ATR in response to replication stress, and DNA metabolic pathways (Figure 2A).

We also analyzed gene expression in DU-145 cells after treatment with DAC and identified 16,092 probes (representing 5851 genes, FDR p<0.05) that showed significantly increased expression in treated cells when compared to untreated cells. There were also 12,970 probes (4067 genes; FDR p<0.05) that showed decreased expression. GSEA analyses of significantly differentially expressed genes in DU-145 cells post-DAC identified the most significant pathways including microtubule motor activity, lysosome pathway, activation of ATR in response to replication stress, and fatty acid oxidation (Figure 2B).

### Correlation between reduced methylation and increased mRNA expression

In order to determine genes controlled by DNA methylation-dependent mechanisms, we correlated regions of CpG hypermethylation in untreated cells with increased gene expression after treatment. We found 670 genes, representing 1641 CpG probes in the 22Rv1 cell line had increased gene expression (adjusted p<0.05) after DAC treatment. This represents a total of 13% (670/4922) genes that had decreased methylation following DAC treatment. Pathway analysis showed that these genes were involved in cell migration and the regulation of DNA methyltransferase activity.

In the DU-145 cells, we found there were 2330 genes with decreased CpG methylation post DAC treatment, with a corresponding increased mRNA expression (adjusted p<0.05) and these were represented by 9910 CpG probes. This represents a total of 28% (2330/8008) genes that had decreased methylation following DAC treatment. Pathway analysis showed that these genes were involved in the regulation of cell-cell adhesion and focal adhesion assembly as well as cellular proliferation and migration.

### Identification of potential methylation markers in tumor tissues

In order to assess the potential role of these genes in the initiation and/or progression of PCa, we assayed methylation status in PCa tumors using CpG island microarray analysis in 20 PCa tumors, 10 cases with Gleason Scores (GS) 6 (3+3) and 10 cases with GS8 (4+4). We assessed methylation status of the 670 genes (1641 CpG probes) that were hypermethylated in 22Rv1 and transcriptionally upregulated after DAC treatment. Among these genes, 117 genes (represented by 170 probes) showed significant (p<0.05) increased methylation in GS8 compared to GS6 tumors. Nine genes, representing potential biomarkers for PCa, are shown in Table 4. Of these nine probes, eight were intragenic while one was located in the promoter region. A number of these genes have been previously shown to be methylated in PCa, including transforming growth factor beta 2 (*TGFB2*), transcription factor AP-2 beta (*TFAP2B*), T-box 2 (*TBX2*), tescalcin (*TESC*), and desmocollin 3 (*DSC3*). We also identified novel potential methylation markers for PCa including actin, alpha 1, skeletal muscle (*ACTA1*), beta-1,4-N-acetyl-galactosaminyl transferase 1 (*B4GALNT1*), nuclear receptor interacting protein 3 (*NRIP3*), and TNF receptor-associated factor 3 (*TRAF3*).

**Table 4:** Genes identified through analysis with 22Rv1 cells that showed significant increased methylation in Gleason score 8 vs Gleason score 6 prostate cancer tumors

Gene Symbol	Gene Name	CpG Location	Fold Change	p- value
*NRIP3*	nuclear receptor interacting protein 3	Promoter	2.023	<0.001
*B4GALNT1*	beta-1,4-N-acetyl-galactosaminyl transferase 1	Intragenic	2.19	<0.001
*ACTA1*	actin, alpha 1, skeletal muscle	Intragenic	3.026	0.0007
*TFAP2B*	transcription factor AP-2 beta (activating enhancer binding protein 2 beta)	Intragenic	2.219	0.0039
*TGFB2*	transforming growth factor, beta 2	Intragenic	1.846	0.0048
*TBX2*	T-box 2	Intragenic	1.558	0.0121
*TESC*	tescalcin	Intragenic	1.545	0.0191
*TRAF3*	TNF receptor-associated factor 3	Intragenic	1.804	0.0205
*DSC3*	desmocollin 3	Intragenic	1.508	0.0481

We performed a similar analysis with genes identified in the DU-145 cells. We identified 835 genes (1456 probes) that showed reduced methylation and increased gene expression post-DAC treatment in cells and had significant differential methylation in GS8 vs. GS6 PCa patients (p<0.05). Eight genes representing potential biomarkers are shown in Table 5. Interestingly, this analysis identified and confirmed a few genes that we previously reported to be associated with aggressive PCa including homeobox D3 (*HOXD3*), T-box 15 (*TBX15)*, and T-box-3 (*TBX3*), as well as genes formerly reported methylated in PCa including homeobox D8 (*HOXD8*) and ventral anterior homeobox 1 (*VAX1*). Furthermore, we identified genes that have not been previously reported to be methylated in PCa including homeobox B6 (*HOXB6*), forkhead box D2 (*FOXD2*) and actin, alpha 1, skeletal muscle (*ACTA1*).

**Table 5:** Genes identified through analysis with DU-145 cells that showed significant increased methylation in Gleason score 8 vs Gleason score 6 prostate cancer tumors

Gene Symbol	Gene Name	CpG Location	Fold Change	p-value
*ACTA1*	actin, alpha 1, skeletal muscle	Intragenic	3.026	0.0007
*TBX3*	T-box 3	Intergenic	2.939	0.0016
*HOXD3*	homeobox D3	Intragenic	2.124	0.0026
*VAX1*	ventral anterior homeobox 1	Intragenic	3.679	0.0051
*TBX15*	T-box 15	Intragenic	3.155	0.0102
*HOXD8*	homeobox D8	Promoter	2.043	0.0124
*HOXB6*	homeobox B6	Intragenic	2.165	0.0174
*FOXD2*	forkhead box D2	Promoter	2.030	0.0243

We validated the correlation of methylation status with Gleason grade using an independent publically available database. Clincopathological and survival data for 127 PCa patients is shown in Supplementary Table 1. We accessed Level 3 methylation data from The Cancer Genome Atlas (TCGA) database, as described in the Materials section, and correlated with Gleason score. We found actin, alpha 1, skeletal muscle (*ACTA1*) had significantly higher methylation in GS ≥8 vs. GS7 patients (p=0.035; Table 6). Also, we found homeobox D3 (*HOXD3*) showed higher methylation levels in GS≥8 vs. GS≤6 tumors (p=0.025) while higher methylation levels of beta-1,4-N-acetyl-galactosaminyl transferase 1 (*B4GALNT1*) were associated with GS ≥8 vs. GS7 patients (p=0.027).

**Table 6:** Association of gene methylation status with Gleason Score

Clinical	χ2 p-value		
Characteristic	*ACTA1*	*HOXD3*	*B4GALNT1*
Gleason Score			
≥8 vs. ≤6	0.258	0.025	0.205
			
≥8 vs. 7	0.035	0.113	0.027
			

**Figure 3:**
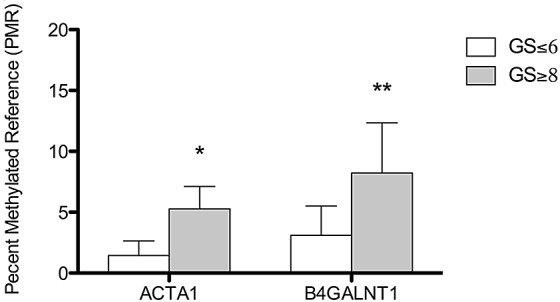
Bar graph showing differential methylation levels in GS≥8 vs GS≤6 prostate tumor specimens *ACTA1* showed significantly higher percent methylated reference (PMR) values in GS≥8 cases (average PMR=5.27) vs. GS≤6 cases (average PMR=1.44). *B4GALNT1* had higher PMR values in GS≥8 tumors (average PMR=8.23) vs GS≤6 cases (average PMR= 3.11). *;p=0.040, **; p=0.005.

We experimentally verified increased methylation of *ACTA1* and *B4GALNT1* in higher Gleason grade tumors in a separate, independent cohort of patients. We measured methylation levels using the MethyLight assay, on an independent cohort of 10 cases of GS≥8 vs. 10 cases of GS≤6 specimens. We found that *ACTA1* showed significantly higher percent methylated reference (PMR) values in GS≥8 cases (average PMR=5.27) vs. GS≤6 cases (average PMR=1.44; p=0.040, Figure 3), which confirmed our previous results. In addition, we found *B4GALNT1* higher PMR values in GS≥8 tumors (average PMR=8.23) vs GS≤6 cases (average PMR= 3.11; p=0.005, Figure 3), again, confirming our previous results.

## DISCUSSION

In this study, we preformed comparative assessment of methylation and gene expression data from PCa cell lines after treatment with the demethylating agent DAC to identify genes that are potentially regulated by DNA methylation dependent mechanisms in PCa. These data provide further insight to the contribution of methylation mechanisms to PCa tumorigenesis. We further explored the role of these genes in PCa carcinogenesis by performing GSEA analysis and determined the biological pathways that are affected by DNA methylation in these cells. Genes regulated by methylation mechanisms also represent potential biomarkers for PCa patients. We validated potential biomarkers using publically available databases and experimentally using two separate cohorts of patients.

In order to identify regions of CpG methylation in PCa cells, we employed a restriction enzyme based differential methylation hybridization strategy that enriched for hypomethylated gDNA [22]. This approach differs from others that focused mainly on the enrichment and detection of hypermethylated DNA [23-26]. Although focusing on the hypermethylated fraction is very useful for detecting major epigenetic changes in some regions of the genome, the overall proportion of interrogated CpG probes is substantially lower compared to approaches that focus on the unmethylated DNA fraction [22]. In addition, since we treated cells with a demethylating agent, we reasoned that quantifying methylation based on the hypomethylated fraction would be more logical to gain further insights into the genome-wide methylation events. Interestingly, we found that even though DU-145 cells had approximately three times more probes with reduced methylation post-DAC treatment than 22Rv1 cells (32,511 vs. 11,212, Figure 1), the locational distribution of the CpG probes was very similar. Approximately 23% CpG probes were found in promoter regions while approximately 71% CpG probes were intragenic, in both cell lines. This implies a conserved function for CpG methylation outside promoter regions. The significance of gene body DNA methylation is not well understood. CpG islands in intragenic or intergenic regions have been shown to exhibit high tissue-specific DNA methylation [27]. Recently, Maunakea et al., [28] showed DNA methylation in gene bodies has a role in regulating cell context-specific alternative promoters. More specifically, they examined the human SHANK3 locus and its mouse homologue and demonstrated that this tissue-specific DNA methylation regulates intragenic promoter activity *in vitro* and *in vivo*. Moreover, intragenic DNA methylation has been shown to have an effect on chromatin structure [29] gene expression [30, 31], and transcriptional elongation [29, 32].

Our integrated analysis identified genes regulated by methylation in PCa cell lines. We examined methylation levels of these genes in PCa patient tumors and found a number of genes showed significantly differential methylation levels in GS8 vs. GS6 tumors, suggesting potential prognostic significance. We identified increased methylation in genes that have been previously reported, including, *HOXD3*, *TBX15*, *DSC3* and *TGFB2* in GS8 vs. GS6 tumors (p<0.01; Table 4 and Table 5) [16, 21, 33-35]. In addition, we identified a number of genes that have not been implicated in PCa previously. *ACTA1* was shown to have increased methylation in GS8 vs GS6 tumors using genome wide methylation profiling (p<0.05; Table 4 and 5), in GS≥8 vs. GS7 tumors using the TCGA database (p=0.035, Table 6), and as well as an independent set of GS≥8 vs. GS≤6 tumors (p<0.05, Figure 3). *ACTA1* is developmentally regulated and one study showed that its promoter methylation was suppressed in mice that were exposed to a diet with high amounts of nutritional phytoestrogens compared to control mice [36]. To the best of our knowledge, this is the only report in the literature examining *ACTA1* methylation in cancer. In addition, we examined *B4GALNT1* methylation levels and found higher methylation was associated with higher Gleason grade in the three independent patient cohorts examined; the TCGA publically available database (p=0.027, Table 6), and two experimentally verified patient tumor cohorts (p<0.001, Table 4; p<0.005, Figure 3). The *B4GALNT1* gene codes for GalNAc-T, a glycosyltransferase, that is involved in the first step of the biosynthesis of all complex derivatives of asialo, a-, b- and c-series gangliosides. Increased mRNA expression of *B4GALNT1* was observed after DAC treatment in colorectal cancer [37] yet this is the first report describing the association of *B4GALNT1* methylation with PCa progression. These markers should be validated in independent patient populations.

Methylation-based testing that would allow for the accurate prediction of disease aggressiveness at the time of prostate biopsy would greatly benefit patients. Patients who have aggressive disease would be treated with radical prostatectomy, while patients predicted to have indolent disease would be treated with a less aggressive course of treatment, such as active surveillance, and would not have to needlessly endure the harsh side effects of radical prostatectomy. Epigenetic testing, by measuring increased methylation levels of three genes, *GSTP1*, *APC* and *RASSF1,* has been shown to accurately predict the presence of PCa in a negative biopsy (ConfirmMDx, MDxHealth, Irvine, CA, USA). Our study provides potential candidate genes for a methylation-based test for PCa aggressiveness. These candidate genes require large-scale validation.

This study provides a comprehensive analysis through combined methylation and gene expression data from cell lines and methylation data from tumor specimens with different Gleason scores to help gain a better understanding of the epigenetic mechanisms that contribute to PCa tumorigenesis. Our analysis identified many novel genes and interesting pathways that were dysregulated in PCa cell lines after treatment with the demethylating agent, which warrant a further in-depth analysis. These genes also represent potential prognostic markers for PCa patients and provide the foundation to a better understanding of the molecular mechanisms involved in PCa.

## MATERIALS AND METHODS

### Cell Lines and Treatment

The human cell lines DU-145 (ATCC# HTB-81) and 22Rv1 (ATCC# CRL-2505) were cultured in RPMI 1640 media supplemented with 10% fetal bovine serum. Cells were maintained in humidified atmosphere with 5% CO_2_ at 37°C. DNA was extracted after harvesting the cells by trypsinization using the QIAamp DNA Mini Kit (Qiagen). Total RNA was extracted using Trizol (Invitrogen) as recommended by the manufacturer.

For treatment with 5-Aza 2′–deoxycitidine (DAC), DU-145 and 22Rv1 cells were plated in 6 cm dishes and incubated in culture medium with 2 μg/mL DAC for 4 days with medium change every 2 days. Since DAC is incorporated into the DNA of dividing cells and inhibits DNA methylation by forming covalent complexes with DNA methyltransferases, it was important to treat the cells at least over their doubling time. To ensure maximum demethylation, we treated the cells over two doubling times. The doubling time for DU-145 and 22Rv1 cells is 29 hrs and 40 hrs, respectively. Cells were harvested and genomic DNA and RNA were extracted as described above.

### Patient Samples

We used two separate cohorts of patient tissues. The first patient cohort consisted of twenty fresh frozen PCa tissue samples [10 Gleason score (GS) 6 (3+3) and 10 GS 8 (4+4)] and were obtained from prostatectomy specimens of patients with prostate cancer (PCa) diagnosed between 2001 and 2007 at the University Health Network (UHN), Toronto. PCa specimens were subjected to histological examination by an expert pathologist (TVDK) to confirm tumor. The second patient cohort consisted of twenty formalin fixed paraffin embedded (FFPE) PCa tissue samples [10 Gleason score (GS) ≤6 and 10 GS 8≥] were obtained from prostatectomy specimens of patients with prostate cancer diagnosed between 2007 and 2011 at the UHN, Toronto. PCa specimens were reviewed by a GU pathologist (TVDK) to confirm Gleason grade. For both cohorts, patients who had therapy prior to surgery were not included in this study. All patients consented to the donation of removed tissue to the UHN tissue bank and samples were obtained according to protocols approved by the Research Ethics Boards of Mount Sinai Hospital and UHN, Toronto, ON.

DNA was collected and extracted as previously described [38]. Briefly, consecutive serial sections (10 micron each) were obtained from FFPE tissues and air-dried onto slides. Areas enriched in tumor cells (>80% neoplastic cellularity) representing each Gleason grade were marked on the slide and were manually micro dissected. Tissues were digested in 30μL proteinase K at 56°C overnight, followed by the addition of 20μL proteinase K and digestion for one hour at 56°C the following day. The recommended protocol for extraction of DNA from FFPE tissue using the QIAmp DNA mini kit (Qiagen) was then followed. DNA concentration was determined using the NanoDrop 2000 (Thermo Scientific, Wilmington, USA) and stored at 4°C.

### Differential Methylation Profiling and CpG Island Microarray

We assayed for genome wide CpG island methylation in cell lines and tumor tissues using methylation array profiling strategy. Methylation status in 22Rv1 and DU-145 prostate cancer cell lines was determined by using differential methylation hybridization approach whereby the hypomethylated fraction was enriched pre (or prior to) and post-DAC treatment. The hypomethylated fraction was isolated by first digesting genomic DNA with the methylation-sensitive restriction enzymes *Hpa*II and *Bst*UI [22]. The cleaved ends were ligated with linkers, followed by further digestion with the restriction enzyme McrBc which cleaves DNA containing methylcytosine on one or both strands and will not digest unmethylated/hypomethylated DNA. Linker PCR reactions were then performed to generate the final target amplicons for microarray hybridization. Only the final hypomethylated amplicons were purified using the QIAquick PCR purification kit (Qiagen). We compared the hybridization patterns before and after DAC treatment in each cell line. Experiments were performed in triplicate.

Differential methylation status was determined by comparing the hypomethylated fraction before and after DAC treatment. Genes that showed higher signal in the hypomethylated fraction post-DAC were considered. Any probes that were not associated with a gene were removed. Probes were considered significant if p<0.05 and retained for further analysis.

CpG methylation status was assessed in tumor tissues by first performing differential methylation hybridization and selecting for the hypermethylated fraction as described previously [38]. Briefly, genomic DNA was digested with *Mse*I. The cleaved ends were ligated with annealed H-12/H-24 linkers, followed by further digestion with two successive rounds of digestion with methylation-sensitive enzymes, *Hpa*II and *Bst*UI. Linker PCR reactions were then performed with pre-treated DNA to generate the final target amplicons for microarray hybridization. Final hypermethylated amplicons were purified using the QIAquick PCR purification kit (Qiagen). The reference sample consisted of DNA isolated from lymphocytes of six healthy men age-matched with PCa patients (GEO accession # GSE15298­). Reference samples were similarly treated for final target generation and pooled amplicons were co-hybridized to the test cases for individual arrays. Methylation status was determined as described previously [38].

### Microarray gene expression profiling and data analysis

Gene expression was assayed by microarray profiling performed on a custom Agilent 4×180 k oligo custom array [39] using triplicates of each of DAC-treated and non-treated cells. The custom microarray incorporates Agilent human gene expression protein-coding probes as well as non-coding probes, with the probes targeting exonic regions, 3′UTRs, 5′UTRs, as well as intronic and intergenic regions. After RNA isolation was performed (RNeasy Mini Kit, Qiagen) samples were analyzed by a Bioanalyzer (Agilent) to en­sure high quality and integrity of RNA samples. 200ng of RNA from each sample was amplified and labeled according to the protocol for One-Color Microarray-Based Gene Expression Analysis (Low Input Quick Amp Labeling Kit, Agilent). The input RNA was reversed transcribed into cDNA, using an oligo-dT-promoter primer, which introduces a T7 promoter region. The subsequent *in vitro* transcription uses a T7 RNA polymerase, which simultaneously amplifies target material and incorporates cyanine 3-labeled CTP. cDNA synthesis was performed at 40°C for 2 h, respectively. The labeled cRNA was purified with RNEasy Mini spin columns (Qiagen) and quantified using a Nanodrop-1000. 1650 ng cRNA from each sample were loaded onto 4×180 k custom microarray (GEO platform accession GPL16604) and allowed to hybridize at 65°C for 17 h. The arrays were scanned with the Agilent Microarray Scanner G2565CA.

Microarray data were processed with Agilent Feature Extraction Software (v10.7.3.1). A quantile between array normalization was applied and differential expression was determined using a Bayesian adjusted t-statistic from a Linear Models for Microarray Data (LIMMA) linear model. Gene expression was considered significant if fold change was >1.25 and p <0.05 (adjusted for a false discovery rate of 5%).

### Gene Set Enrichment Analysis (GSEA)

Pathway enrichment analyses were performed on 22Rv1 and DU-145 cell line expression array datasets (DAC treated) to determine pathways or biological processes over-represented in genes with increased or decreased expression following DAC treatment. Two gene lists were generated for pathway enrichment analyses by identifying unique genes within each expression dataset (treated 22Rv1 and DU145 cells). Genes were pre-ranked according to the p-value and fold-change associated with differential expression [-log(p-value)*SIGN(fold-change)]. Genes with p-value less than 0.001 were assigned equal ranking. Further, in cases where distinct genes indicated multiple significant probes, the greatest absolute value was taken. The software used for pathways enrichment analysis was Gene Set Enrichment Analysis (GSEA) [40, 41]. Each gene list was uploaded into GSEA and the program was run with 1000 gene set permutations to calculate false discovery rate using multiple hypothesis testing. Downloaded gene sets [http://download.baderlab.org/EM_Genesets/September_14_2013/Human/symbol/] were used to discover over-represented pathways. GSEA enrichment results were shown using normalized enrichment scores (NES), which is a value assigned to each gene set after normalization across all analysed gene sets. NES is calculated by the following formula: NES = actual ES/mean (ESs against all permutations of the dataset), and is recommended as the primary statistic to examine GSEA results.

### Prognostic significance and statistical analyses

In order to assess the prognostic ability of methylated genes, we queried the cBio Cancer Genomics Portal (www.cbioportal.org/publicportal/) and The Cancer Genome Atlas (TCGA) database for methylation data in prostate adenocarcinoma patients. Methylation β-values for identified genes of 248 patients with primary PCa using ‘Level 3′ methylation data [normalized gene expression data derived from the Cancer Genome Characterization Center (CGCC) at the University of North Carolina (unc.edu) using the Illumina Infinium Human Methylation 450 platform] were accessed. Clinical information and survival data were obtained from The Cancer Genome Atlas (TCGA), available through the cBio Cancer Genomics Portal (www.cbioportal.org/publicportal/). Clinical data was available for 124 patients (Supplementary Table 1). We correlated methylation levels with Gleason score using the Chi-squared test.

### MethyLight Analysis

DNA methylation analysis was performed on PCa tumor specimens recruited from UHN using the semi-quantitative MethyLight assay [42]. Briefly, 20ng of bisulfite-converted genomic DNA was amplified using locus specific PCR primers flanking an oligonucleotide probe with a 5′ fluorescent reporter dye and a 3′ quencher dye. Primers and probe sequences used for the target genes, *ACTA1* and *B4GALNT1,* and the reference gene, *ALU-C4* are shown in Table 1. Methylation levels were assessed by the Percent Methylated Reference (PMR), which is calculated by dividing the *target gene:Alu-C4* ratio of a sample by the *target gene:Alu-C4* ratio of commercially available fully methylated DNA (Millipore) and multiplying by 100. The *Alu-C4* PCR products (generated from a consensus CpG-devoid region of the *ALU* repetitive element) were used as controls to normalize for input DNA. Samples were analyzed using the ABI 7500 RT-PCR thermocycler [43]. Cycling conditions were as follows; 95°C for 10 minutes, followed by 50 cycles of 95°C for 15 sec and 60°C for 1 minute. Samples were analyzed in duplicate.

## SUPPLEMENTARY MATERIAL TABLE


